# Magneto-optical heterostructures with second resonance of transverse magneto-optical Kerr effect

**DOI:** 10.1038/s41598-024-54039-3

**Published:** 2024-02-12

**Authors:** Amene Rezaeian, Mahmood Hosseini Farzad

**Affiliations:** Department of Physics, College of Sciences, Shiraz University, Shiraz, 71946-84795 Iran

**Keywords:** Magneto-optics, Nanophotonics and plasmonics

## Abstract

Two conventional magneto-plasmonic (MP) structures are firstly superimposed with mirror symmetry to form a symmetric MP heterostructure. These two MP components are separated from each other by a noble metallic layer. The unique feature of this novel heterostructure is that both magneto-plasmon modes of the up and down MP portions can be coupled as the spacer becomes thinner. This intertwining effect leads to appearance of a new peak in the angular transverse magneto-optical Kerr effect (TMOKE) curve of the heterostructure. This new peak which is reported for the first time in the TMOKE signal, is generally similar to plasmon induced transparency (PIT) phenomenon observed in plasmonic multilayered structures. We entitle this novel effect as “second resonance of TMOKE signal”. More importantly, the occurrence angle and magnitude of the second peak can be controlled by varying the thickness and material of separating layer between two MP parts. Also, the dispersion diagram of the heterostructure shows this coupling so that two branches convert into four branches by reducing the thickness of spacer. Furthermore, coupled oscillators model confirms emergence of the second peak in the TMOKE signal. These results can offer great promise for increasing sensitivity of conventional magneto-optical refractive index sensors.

## Introduction

Surface plasmon polariton (SPP) modes are interesting effects which are resulted from the interaction between incident electromagnetic waves and oscillations of the electron plasma located on a metal-insulator interface. The unique capability of SPPs in the incarceration of electromagnetic energy at sub-wavelength scale caused that they have been employed in various applications especially in the plasmonic sensors^1–6^. One type of plasmonic systems containing two semi-infinite metallic layers that separated from each other by thin dielectric layer named Metal-Insulator-Metal (MIM) structures^7–10^. When the thickness of dielectric layer is sufficiently large, it behaves like a planar waveguide (WG) and allows the propagation of electromagnetic waves. In this case, when the peak of narrow resonances relevant to the SPPs excitation and the peak of broad resonances relevant to the WG modes occurred in an identical incident angle, one can observe a peak in the angular reflectivity curve, instead of minimum reflection in conventional plasmonic structure. This phenomenon is named plasmon induced transparency (PIT) effect^11^, borrowed from electromagnetically induced transparency (EIT) effect which is firstly observed in quantum mechanical systems and later in other physical systems^12–17^. When the thickness of dielectric layer changes, PIT can convert into fano resonance. In this case, the angle of the resonance peak of WG mode is slightly smaller or larger than that of the SPP resonance peak^11^. PIT and fano resonance can enhance the sensitivity of standard plasmonic sensors^18–20^.

By inserting a ferromagnetic layer into the plasmonic systems, magneto-plasmonic (MP) structures to be formed^21–23^. In this situation an applied magnetic field can influence the SPP properties^24,25^. This effect was first investigated theoretically in metals^26^ and semiconductor-based SPPs^27–29^. On the other hand, the SPP excitations also affected the magneto-optic (MO) properties of the magnetized layer in these MP structures. Both magneto-optical Kerr and Faraday effects are enhanced in MP structures^21–23,30–33^. MP structures are suitable condidates for improving the sensing properties of sensors^34–40^. Similar interacting resonant phenomena like PIT and fano resonance have been observed in MO activity of MP structures^41,42^. In the Faraday effect as reported in the literature^41^, the longitudinal magnetized layer can increase the transparency of a trilayer magneto-plasmonic crystal by 24% at $\lambda =840\, \text{nm}$. In other report a purely plasmonic disk interacts with a magneto-plasmonic superlattice one by an electric dipole which in turn produced by an applied magnetic field perpendicular to the disks. This interaction leads to induced MO activity in the plasmonic disk. Furthermore, at certain wavelengths and thicknesses of the separating layer between these two components the interaction is canceled. This leads to strong reduction in MO activity of the whole system. This inhibition of the resulting MO activity constitutes MO counterpart of the EIT effect^42^.

In this paper, we numerically demonstrate a new peak that is appeared in the angular transverse magneto-optical Kerr effect (TMOKE) curve of symmetric MP heterostructure. This heterostructure contains two identical conventional MP structures which are separated from each other by a noble metallic layer. The new peak is created at the specific incident angle which both MP portions strongly interact with each other. The occurrence angle and magnitude of the new peak strongly depend on the thickness and material of the spacer. Also, coupled oscillators model provides main evidences of the coupling between MO activities of the two MP components which leads to appearance of the second peak in the TMOKE signal of proposed heterostructure.

## Results

Here, we focus our attention on the TMOKE configuration. In this case, the magnetization vector is perpendicular to the SPP wave vector and parallel to the interface of the mediums. Presence of an external magnetic field causes that dielectric permittivity of magnetic materials becomes a tensor as follows^43^:

1 $$\begin{aligned} \varepsilon = \begin{bmatrix} \varepsilon _{m} &{} 0 &{} ig\\ 0 &{} \varepsilon _{m} &{} 0\\ -ig &{} 0 &{} \varepsilon _{m} \end{bmatrix}, \end{aligned}$$

where $\varepsilon _{m}$ is permittivity and *ig* is proportional to magnetization. In this configuration no change in the polarization of the incident light is occurred however the reflected intensity is changed by the incident angle of p-polarized light. The TMOKE signal, $\Delta R/{R}$, is defined as:

2 $$\begin{aligned} \frac{\Delta R}{R}=\frac{R_{pp}(+H)-R_{pp}(-H)}{R_{pp}(H=0)}, \end{aligned}$$

where $R_{pp}(H=0)$ and $R_{pp}(\pm H)$ are reflectivities in the absence and presence (± directions) of the external magnetic field, respectively.

**Figure 1:**
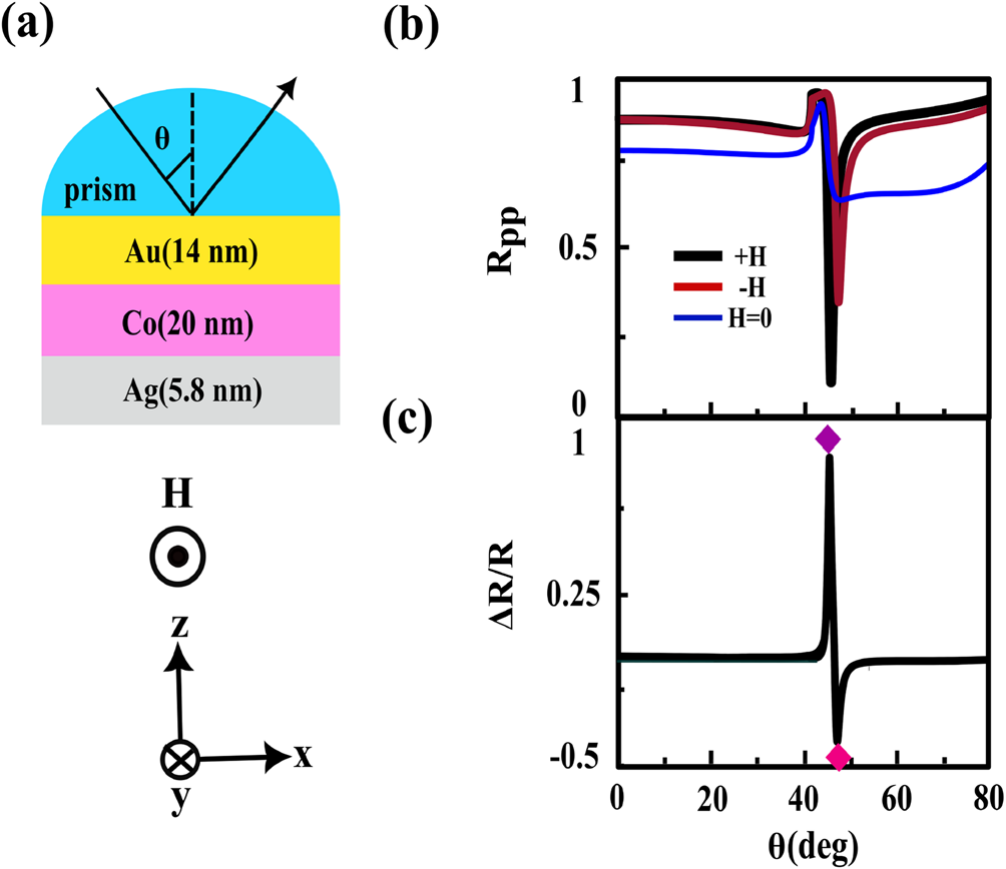
(**a**) schematic of the conventional trilayer MO structure in the kretschmann configuration, (**b**) and (**c**) parts show the angular dependences of the $R_{pp}(\pm H, H=0)$, and their resulting TMOKE signal, respectively. The violet and pink diamonds correspond to the maximum and the minimum of TMOKE signal, respectively where in the field distributions will be calculated (see Fig. 5 in the following).

**Figure 2:**
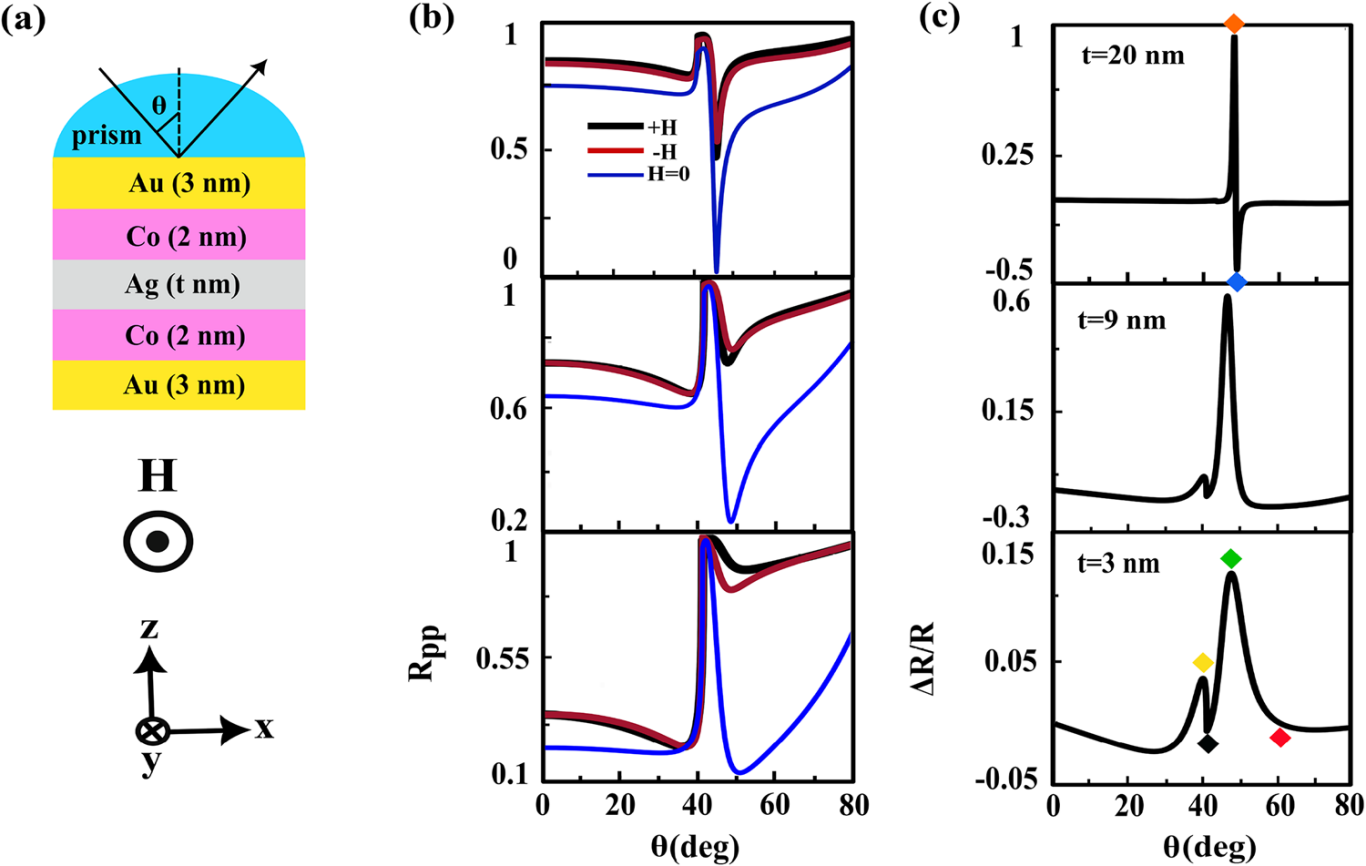
(**a**) schematic of the offered MO heterostructure, the angular dependences of $R_{pp}(\pm H, H=0)$ and their resulting TMOKE signal are shown by (**b**) and (**c**) parts, respectively for three thicknesses of Ag layer.The colored diamonds appearing in the TMOKE signal for 20 nm and 3 nm thicknesses of Ag layer correspond to the angular positions which are used later in calculation of the field distributions (see the following Figs. 3 and 4).

First of all, we consider the conventional trilayer MO structure which is containing Au (14 nm), Co (5.7 nm) and Ag (5.8 nm) layers, respectively from up to down over the semi-cylindrical BK7 prism, see Fig. 1a. After this introduction, we propose a MO heterostructure with two ferromagnetic layers which is formed by adding another MP (Co/Au) layer to this conventional MO structure, see Fig. 2a. All the thicknesses of different layers are designed and justified. In order to simulate these structures by COMSOL Multiphysics we utilized the values of $n_{prism}=1.5151$, $n_{Au}=0.196+i3.255$ and $n_{Ag}=0.134+i3.986$^11^ for refractive indices of prism, Au and Ag, respectively at incident wavelength of 632.8 nm. Also, the dielectric constant of the Co film is considered as $\varepsilon _{m}=-10.6+i24.3$ and its MO constant is $g=0.2-i0.944$^44^, respectively. The $R_{pp}(H=0)$ and $R_{pp}(\pm H)$ curves versus incident angle are plotted in Fig. 1b and the top frames of Fig. 2b for the conventional and offered structures, respectively. We used these information for producing the TMOKE signal [see Eq. (2)] of each structure, see Fig. 1c and the top portion of Fig. 2c. By comparing these figures, It is easy to see that the TMOKE signal for proposed heterostructure became similar to that of for conventional trilayer MO structure. Now, we gradually reduce the thickness of the Ag layer. The angular reflectivity curves and their resulting TMOKE signals for 9 nm and 3 nm thicknesses of Ag spacer are shown in the middle and bottom frames of Fig. 2b and c, respectively. As the thickness of Ag layer decreases both MP portions, above (Au/Co) and below (Co/Au) this layer, strongly interact with each other and their magneto-plasmon modes are coupled to each other. As a consequent of this process, a new peak appears in the angular TMOKE curve respect to the corresponding one for this structure with thick Ag layer (20 nm), see Fig. 2c. According to Fig. 2c, bottom frames, the new (second) peak in the TMOKE signal is occurred at the same angle at which the transition from maximum to minimum reflectivity take placed, see the corresponding frames in Fig. 2b. However in the conventional trilayer MO structures^39,40,45^ the angular location of the maximum value of TMOKE signal is the same as the angle in which the minimum reflectivity is occurred. The new peak appeared in the TMOKE signal also occurs in the transition from the maximum and the minimum of TMOKE response of the heterostructure with thick Ag layer (20 nm), see Fig. 2c. We introduce this increasing in the MO activity as a “second resonance of TMOKE signal effect”. According to the above discussion, it can be said that the physical origin of this effect is due to the interaction between both MP parts and coupling their magneto-plasmon modes. To elucidate this process, let us calculate the skin depth $\delta ={1}/{k_{m}}$^46^ of the SPP mode at the first Au/Co interface in the absence and presence of the external magnetic field. For this task, we initially need to obtain the in plane (in x-direction) and out of plane (in z-direction) components of SPP wave vector at this interface. The former is given by:

3 $$\begin{aligned} \beta (i)=k_{0} n_{prism} sin\theta _{SPP} (i), \quad i=\pm H, H=0 \end{aligned}$$

where $k_{0}$ is the wave vector of the incident light and $\theta _{SPP}$ is the incident angle at which the reflectivity becomes minimum. According to the bottom portion of Fig. 2b we extract three angles of SPP excitation as $\theta _{SPP}(+H)=51.83^{\circ }$, $\theta _{SPP}(-H)=49.29^{\circ }$ and $\theta _{SPP}(H=0)=51.08^{\circ }$. Then by using of Eq. (3) we obtain their corresponding SPP wave vectors as $\beta (+H)\simeq 0.0118 (1/\text{nm})$, $\beta (-H)\simeq 0.0114 (1/\text{nm})$ and $\beta (H=0)\simeq 0.0117 (1/\text{nm})$. For the other component of SPP wave vector one can be used^47^

4 $$\begin{aligned} k_{m}(i)=\sqrt{ \beta ^{2}(i)-k_{0}^{2}(\frac{\varepsilon _{m}^{2}-g^{2}}{\varepsilon _{m}}}), \quad i=\pm H \end{aligned}$$

**Figure 3:**
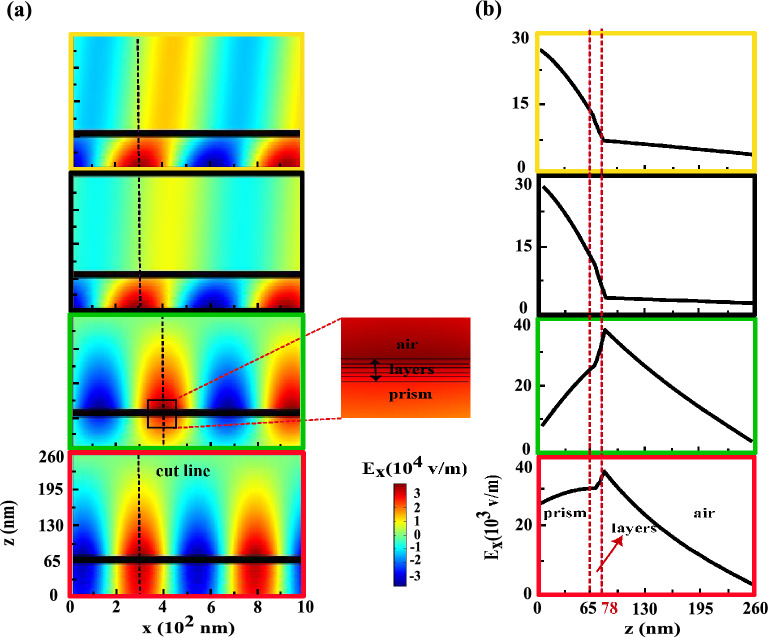
(**a**) near field distributions of the $E_{x}$ component and (**b**) their variations versus z position at the cut lines for the first maximum ($40.26^{\circ }$), first minimum ($41.31^{\circ }$), second maximum ($47.95^{\circ }$) and second minimum ($62.21^{\circ }$) from up to down, respectively. The colors around each frame is in accord to the yellow, black, green and red diamonds in the TMOKE curve in the bottom frame of Fig. 2c. The inset magnifies the details of the investigated heterostructure.

When the external magnetic field is zero $g=0$, Eq. (4) is reduced to the corresponding plasmonic interface, $k_{m}=\sqrt{ \beta ^{2}-k_{0}^{2} \varepsilon _{m}}$^46^. Therefore, we calculate $k_{m}$ for these cases as $k_{m}(+H)\simeq k_{m}(H=0)\simeq 0.03\,(1/\text{nm})$ and $k_{m}(-H)\simeq 0.032\,(1/\text{nm})$. Finally the corresponding skin depths are $\delta (+H)\simeq \delta (H=0)\simeq 33.33\,(\text{nm})$ and $\delta (-H)\simeq 31.25\,(\text{nm})$. By comparing total thickness ($t_{total}=13\,\text{nm}$) of the proposed heterostructure and $\delta$, it is clear that $\delta >t_{total}$. Therefore, the excited SPPs at the first Au/Co interface can be tunneled through the structure and induced the SPPs at the second one. This penetration provides the interaction between both MP branches in this heterostructure.  In order to better understand this phenomenon we try to describe the behavior of the near field distributions of the x-component of the electric field, $E_{x}$, and their variations versus z position (at the specific x positions where in the electric field has its maximum value that are shown by the cut lines) of the layers for heterostructure with 3 nm thickness of Ag layer (investigated heterostructure), see Fig. 3.

**Figure 4:**
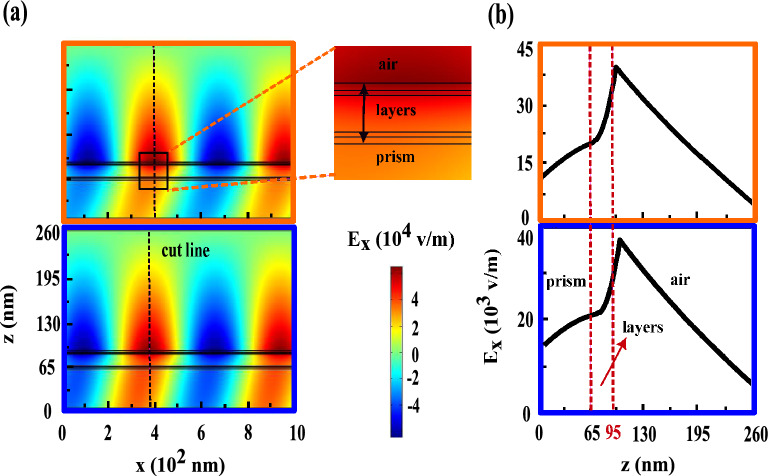
The orange and blue frames show the near field distributions of the $E_{x}$ component (**a**) and their variation versus z position which are plotted at the cut lines (**b**) for the maximum ($47.5^{\circ }$) and the minimum ($48.37^{\circ }$) correspond to the orange and blue diamonds in the top frame of Fig. 2c, respectively. The inset shows the zoom-in the detail of its corresponding structure.

**Figure 5:**
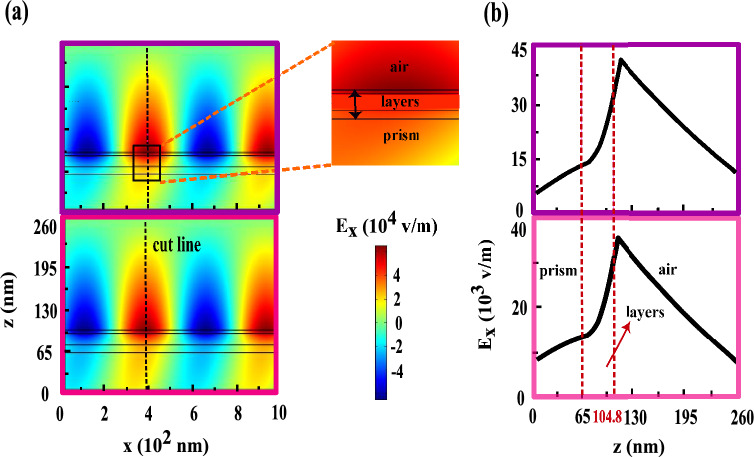
(**a**) near field distributions of the $E_{x}$ component and (**b**) their variations versus z position are plotted at cut lines for the angles $45.37^{\circ }$ and $47.09^{\circ }$ correspond to the maximum and the minimum of TMOKE signal for the conventional trilayer MO structure which are shown by the violet and pink diamonds in Fig. 1c, respectively. The detail of this structure is magnified by the inset.

These quantities are calculated for the first maximum ($40.26^{\circ }$), first minimum ($41.31^{\circ }$), second maximum ($47.95^{\circ }$) and second minimum ($62.21^{\circ }$) which are indicated by yellow, black, green and red diamonds, respectively in the bottom frame of Fig. 2c. At the first maximum (yellow frames), most of the electric field is accumulated inside the prism and a few field transmitted through the structure and after refracted at Au/air interface propagated without appreciable reduction. Since the angle of first maximum is smaller than the critical angle at the prism/Au interface, see the bottom frame of Fig. 2b, thus SPPs never to be excited here. At the first minimum (black frames), a very weak evanescent field is generated at prism/Au interface and in turns after penetration is observed at Au/air interface with opposite direction. Since the phase matching condition between incident light and SPPs does not occur and SPPs can not excite at Au/Co interface nearly all of field is trapped in the prism. At the second maximum (green frames), the excited SPPs at the first Au/Co interface tunneled through the structure and induce the SPPs at the second one and both strongly couple with each other. The interference between re-emitted light from these coupled SPPs and the reflected light from the prism/Au interface leads to considerable enhancement of field at Au/air interface. As a consequent of this process second peak appears at TMOKE signal. Finally due to the angle of second minimum is larger than the angle of SPP excitation, the phase matching condition between incident light and SPPs becomes weak. This causes that the field enhanced at the prism/Au interface, see the red frames. Also, these calculating quantities for thick Ag layer and conventional trilayer MO structures are shown in Figs. 4 and 5, respectively. As you can see in Figs. 4 and 5, there is a basic similarity between the features of these structures. But by inspection in the green frames of Fig. 3, the orange frames of Fig. 4 and the violet frames of Fig. 5 it can be understood that the nature of second peak is completely different from both other TMOKE peaks.

### Explanation of second resonance of TMOKE by dispersion relation of the investigated heterostructure

**Figure 6:**
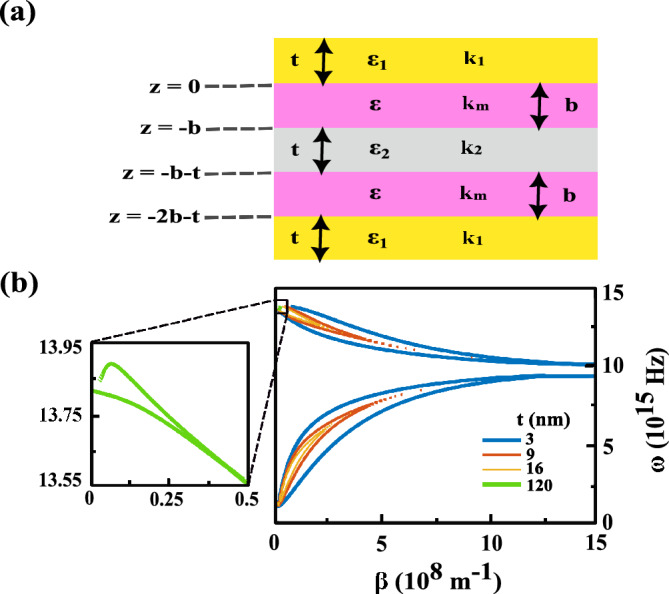
(**a**) schematic of our interested heterostructure with different regions. $\varepsilon _{1}$, $\varepsilon$ and $\varepsilon _{2}$ are dielectric constants of Au, Co and Ag layers, respectively. $k_{1}$, $k_{m}$ and $k_{2}$ are their correspoding z-component wave vectors. t and b are thicknesses of the noble metallic and Co layers which are equal to 3 nm and 2 nm, respectively in our calculations. (**b**) dispersion diagrams for the proposed heterostructure with 3 nm, 9 nm, 16 nm and 120 nm of Ag layer thicknesses. The inset magnifies the dispersion curve of the heterostructure when the thickness of Ag layer is equal to 120 nm.

Now we are interesting to apply some mathematical manipulation in order to obtain the characteristic equation of this MO heterostructure. For this task we consider the following regions $z>0$, $0>z>-b$, $-b>z>-b-t$, $-b-t>z>-b-2t$ and $-2b-t>z$, see Fig. 6a, and obtain the spatial distribution of the electromagnetic field of the SPP associated with each of them. The magnetic fields are defined as

5 $$\begin{aligned}{} & {} H_{y}=A_{1}e^{i\beta x}e^{-k_{1}z}, \end{aligned}$$

6 $$\begin{aligned}{} & {} H_{y}=e^{i\beta x}(A_{2}e^{+k_{m}z}+A_{3}e^{-k_{m}z}), \end{aligned}$$

7 $$\begin{aligned}{} & {} H_{y}=e^{i\beta x}(A_{4}e^{k_{2}z}+A_{5}e^{-k_{2}z}), \end{aligned}$$

8 $$\begin{aligned}{} & {} H_{y}=e^{i\beta x}(A_{6}e^{+k_{m}z}+A_{7}e^{-k_{m}z}), \end{aligned}$$

9 $$\begin{aligned}{} & {} H_{y}=A_{8}e^{i\beta x}e^{k_{1}z}. \end{aligned}$$

The corresponding electric fields can be obtained through Maxwell’s equations (see Supplementary). The continuity of $H_{y}$ and $E_{x}$ at the boundaries of $z=0$, $z=-b$, $z=-b-t$ and $z=-2b-t$ leads to the $8\times 8$ matrix (see Supplementary). To have a nontrivial solution, the determinant of the coefficients matrix must be zero that yields the following SPP dispersion relation

10 $$\begin{aligned} tanh(k_{2}t)=-2\frac{\alpha \sigma }{(\alpha ^{2}+\sigma ^{2})}, \end{aligned}$$

where the parameters $\alpha$ and $\sigma$ are defined as

$$\begin{aligned} {\left\{ \begin{array}{ll} \alpha & =\frac{k_{2}}{\varepsilon _{2}} (1+ \frac{k_{1}}{\varepsilon _{1}}\frac{\varepsilon ^{2}_{m}-g^{2}}{k_{m}\varepsilon _{m}+g\beta }tanh(k_{m}b)\\ \sigma & =\frac{k_{1}}{\varepsilon _{1}} (1+ \frac{\varepsilon _{1}}{k_{1}}\frac{k_{m}\varepsilon _{m}+g\beta }{\varepsilon ^{2}_{m}-g^{2}}tanh(k_{m}b)). \end{array}\right. } \end{aligned}$$

Figure 6b shows the dispersion diagram of the investigated heterostructure which is obtained by substituting the quantities of $\varepsilon _{i}=1-\frac{\omega ^{2}_{pi}}{\omega ^{2}}, k_{i}=\sqrt{\beta ^{2}-\frac{\omega ^{2}}{c^{2}}\varepsilon _{i}} \quad i=1, 2$, $\varepsilon _{m}=1-\frac{\omega ^{2}_{pm}}{\omega ^{2}}$, $k_{m}=\sqrt{ \beta ^{2}-\frac{\omega ^{2}}{c^{2}}(\frac{\varepsilon _{m}^{2}-g^{2}}{\varepsilon _{m}}})$ and $g=\frac{\omega _{pm}^{2}\omega _{c}}{\omega (\omega ^{2}+\omega _{c}^{2})}$ into Eq. (10). Where $\omega _{p1}$, $\omega _{p2}$ and $\omega _{pm}$ are the plasma frequencies of the Au, Ag and Co layers, respectively. $\omega _{c}=eB/m$ is the cyclotron frequency. e and m are the charge and mass of electron. B is the external magnetic field. The basic four dispersion curves correspond to the SPP excitation at the Au/Co, Co/Ag, Ag/Co and Co/Au interfaces of the heterostructure, from up to down, respectively. As it can be observed in Fig. 6b, by increasing the thickness of Ag layer the coupling between upper and lower SPPs decreases. For a thickness of 120 nm, this coupling is completely canceled and two upper branches of dispersion curve remain. These behaviors confirm the results obtained from the simulations discussed above.

In the limit as $\beta$ goes to infinity, Eq. (10) is simplified to the following equation

11 $$\begin{aligned} (\varepsilon _{1}+\varepsilon _{m}-g)(\varepsilon _{2}+\varepsilon _{m}-g)=0. \end{aligned}$$

After substituting the parameters $\varepsilon _{1}$, $\varepsilon _{2}$, $\varepsilon _{m}$ and *g* in Eq. (11), it splits into two equations as follow

12 $$\begin{aligned} \begin{aligned} 2\omega ^{4}+\omega ^{2}(2\omega _{c}^{2}-\omega _{p1}^{2}-\omega _{pm}^{2})-\omega \omega _{c}\omega _{pm}^{2}-\omega _{c}^{2}(\omega _{p1}^{2}+\omega _{pm}^{2})=0\\ 2\omega ^{4}+\omega ^{2}(2\omega _{c}^{2}-\omega _{p2}^{2}-\omega _{pm}^{2})-\omega \omega _{c}\omega _{pm}^{2}-\omega _{c}^{2}(\omega _{p2}^{2}+\omega _{pm}^{2})=0 \end{aligned} \end{aligned}$$

As it is shown from Fig. 6b, each two branches of the dispersion curve merge to each other and consequently only two horizontally lines are observed for very large value of $\beta$. These lines are obtained from the solutions of Eq. (12).

### Justification of second resonance of TMOKE signal by classical coupled oscillators model

**Figure 7:**
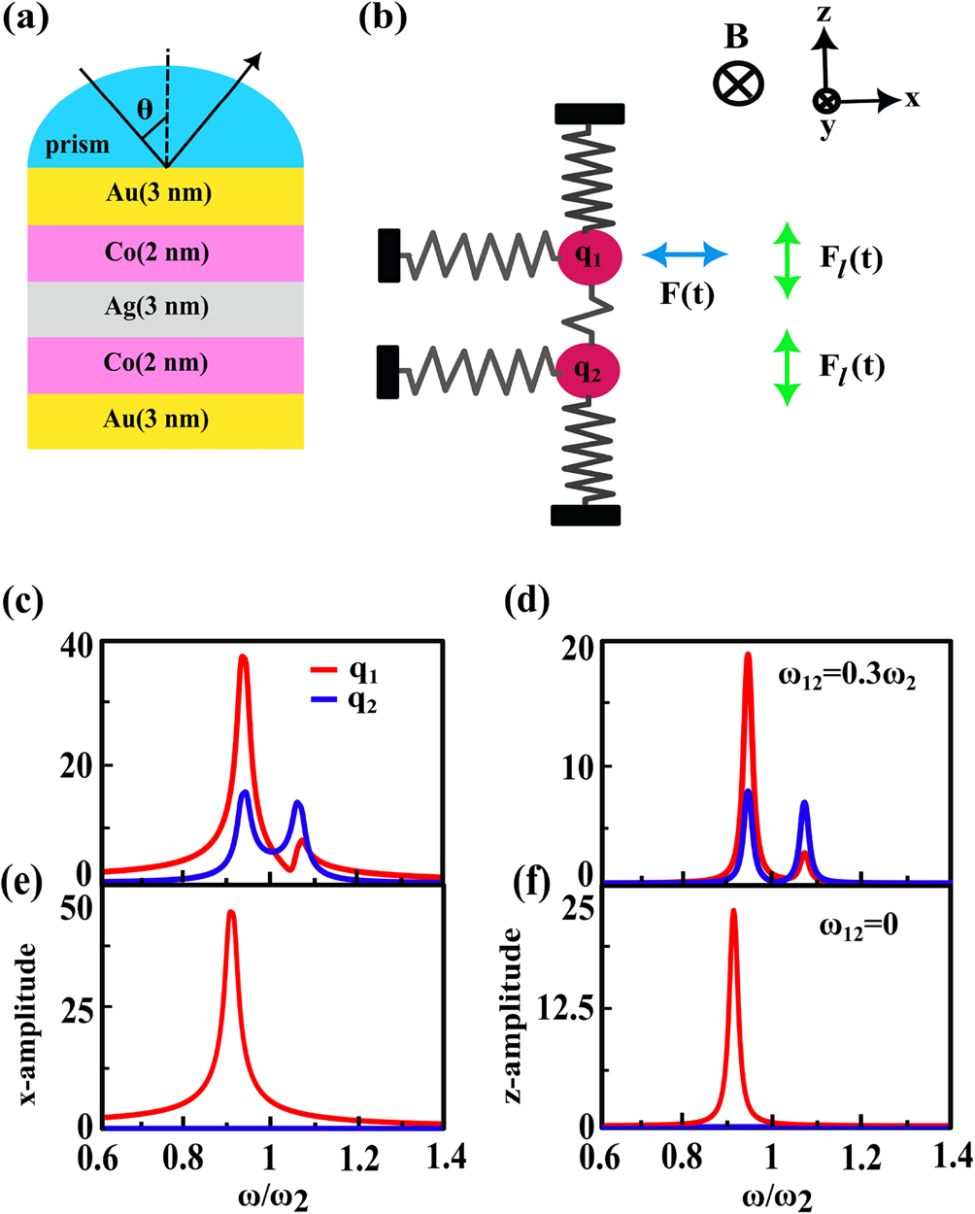
One-to-one correspondence between the investigated MO heterostructure (**a**) and the coupled oscillator model (**b**). The external force *F*(*t*) drives $q_{1}$ and an applied magnetic field induces the Lorentz force $F_{l}(t)$ that oscillates both $q_{1}$ and $q_{2}$. (**c**) and (**d**) are oscillation amplitudes versus the frequency of particles along x- and z-directions for the $\omega _{12}=0.3\omega _{2}$ whereas (**e**) and (**f**) are corresponding quantities for the $\omega _{12}=0$.

As we know to elucidate the mechanism of the PIT and fano resonance effects which are occurred in the interacting resonant systems, coupled oscillators model is usually a suitable approach^17,20,48–50^. In the literature reports^42^, researchers use this mechanical model to discuss something like these effects for the MO activity in the MP nanostructures, with two interacting resonant branches one is purely plasmonic and other is magneto-plasmonic, by considering a neutral mass coupled with a charged mass in the static magnetic field. Accordingly, we utilize this classical model to explain the mechanism of second peak appeared in TMOKE signal. Because our investigated heterostructure has two MP branches,Au/Co and Co/Au, we consider both masses to be charged. In this situation we modeled the first MP portion (Au/Co) and the second one (Co/Au) by charged particles $q_{1}$ and $q_{2}$, respectively. Both of them are in a static magnetic field. Coupling (Ag) layer between them is also denoted by a weak spring as schematically shown in Fig. 7. Placing a mass with charge q in an external magnetic field leads to the appearance of a Lorentz force $F_{l}(t)=q\dot{r}\times B$. Here, due to TMOKE configuration the applied magnetic field (in the y-direction) is perpendicular to the driving external force *F*(*t*) (in the x-direction), where interacts with $q_{1}$ and oscillates it. Then the Lorentz force (in the z-direction) is perpendicular to both driving external force and magnetic field such that the plane of movement of the masses is restricted to the x-z plane. The coupled equations of motion in this model are written as follow:

13 $$\begin{aligned} \begin{aligned} m_{1}\ddot{r_{1}}&=F-k_{1}r_{1}-k_{12}(r_{1}-r_{2})-\beta _{1}\dot{r_{1}}\\ m_{2}\ddot{r_{2}}&=-k_{2}r_{2}-k_{12}(r_{2}-r_{1})-\beta _{2}\dot{r_{2}}, \end{aligned} \end{aligned}$$

where the particle positions $r_{1}$ and $r_{2}$ are referred to their equilibrium state and restricted to the x-z plane, $k_{1}$ and $k_{2}$ are spring constants, $m_{1}$ and $m_{2}$ are the masses of the particles and $k_{12}$ denotes the interaction between them. F is the driving external force which acts on the $q_{1}$. It is important to note that in the presence of magnetic field the damping terms $\beta _{i}$ become tensors:

14 $$\begin{aligned} \beta _{i}=b_{i}+F_{L}^{i}= \begin{bmatrix} b_{i} &{} 0 \\ 0 &{} b_{i} \end{bmatrix}+ \begin{bmatrix} 0 &{} q_{i}B \\ -q_{i}B &{} 0 \end{bmatrix}, \quad i=1, 2 \end{aligned}$$

when the external magnetic field is zero and there is only the friction force in a homogeneous medium, $\beta$ can be considered as a scalar term *b* for each masses. The Lorentz force is written by:

15 $$\begin{aligned} F_{l}^{i}=q_{i}B \begin{bmatrix} 0 &{} 1 \\ -1 &{} 0 \end{bmatrix} \begin{pmatrix} \dot{r_{x}}\\ \dot{r_{z}} \end{pmatrix}. \end{aligned}$$

We assume that the temporal part of the external driving force changes oscillatory ($e^{-i\omega t}$) and $m_{1}=m_{2}=m$. We also consider the frequency relations as below:

16 $$\begin{aligned} \begin{aligned} \omega _{i}^{2}&\equiv \frac{k_{i}}{m},\\ \omega _{12}^{2}&\equiv \frac{k_{12}}{m},\\ \Gamma _{i}&\equiv \frac{\beta _{i}}{m}= \begin{pmatrix} \gamma _{i} &{} \omega _{ci} \\ -\omega _{ci} &{} \gamma _{i} \end{pmatrix}, \end{aligned} \end{aligned}$$

where $\gamma _{i}=b_{i}/m$ and $\omega _{ci}=q_{i}B/m$. In deriving equations of motion in frequency space, we substitute Eq. (16) into Eq. (13), thus:

17 $$\begin{aligned} \begin{aligned} (\omega ^{2}-\omega _{1}^{2}+i\omega \Gamma _{1}-\omega _{12}^{2})r_{1}+\omega _{12}^{2}r_{2}&=-f\\ (\omega ^{2}-\omega _{2}^{2}+i\omega \Gamma _{2}-\omega _{12}^{2})r_{2}+\omega _{12}^{2}r_{1}&=0 \end{aligned} \end{aligned}$$

where $f\equiv F/m$. In matrix form we have:

18 $$\begin{aligned} \begin{pmatrix} \begin{pmatrix} \Omega _{1}^{2}-\omega _{12}^{2} &{} i\omega \omega _{c1} \\ -i\omega \omega _{c1} &{} \Omega _{1}^{2}-\omega _{12}^{2} \end{pmatrix}&{} \begin{pmatrix} \omega _{12}^{2} &{} 0\\ 0 &{} \omega _{12}^{2} \end{pmatrix}\\ \begin{pmatrix} \omega _{12}^{2} &{} 0\\ 0 &{} \omega _{12}^{2} \end{pmatrix} &{} \begin{pmatrix} \Omega _{2}^{2}-\omega _{12}^{2} &{} i\omega \omega _{c2} \\ -i\omega \omega _{c2} &{} \Omega _{2}^{2}-\omega _{12}^{2} \end{pmatrix} \end{pmatrix} \times \begin{pmatrix} r_{1}\\ r_{2} \end{pmatrix}= \begin{pmatrix} -f\\ 0 \end{pmatrix}, \end{aligned}$$

where $\Omega _{i}^{2}=\omega ^{2}-\omega _{i}^{2}+i\omega \gamma _{i}$. Here, since the first MP portion (Au/Co) of the investigated heterostructure is similar to the second one (Co/Au) we assume that $\omega _{c1}=\omega _{c2}$ and $\gamma _{1} =\gamma _{2}$. The solutions of Eq. (18) can be written as follow:

19 $$\begin{aligned} {\left\{ \begin{array}{ll} x_{1}=\frac{b}{a}\\ z_{1}=\frac{ic}{a}\small, \\ x_{2}=\frac{d}{a}\\ z_{2}=\frac{ie}{a} \end{array}\right. } \end{aligned}$$

where *a*, *b*, *c*, *d* and *e* parameters are defined as: $${\left\{ \begin{array}{ll} a=2\omega _{12}^{2}(-\Omega _{1}\Omega _{2}(\Omega _{1}+\Omega _{2})+\omega ^{2}(\omega _{c1}^{2}\Omega _{2}+\omega _{c2}^{2}\Omega _{1})) +(\omega ^{2}\omega _{c1}^{2}-\Omega _{1}^{2})(\omega ^{2}\omega _{c2}^{2}-\Omega _{2}^{2})+\omega _{12}^{4}((\Omega _{1}+\Omega _{2})^{2}-\omega ^{2}(\omega _{c1}+\omega _{c2})^{2}) ,\\ b=-\omega _{12}^{4}(\Omega _{1}+\Omega _{2})+\Omega _{1}(\omega ^{2}\omega _{c2}^{2} -\Omega _{2}^{2})+\omega _{12}^{2}(\Omega _{2}(2\Omega _{1}+\Omega _{2})-\omega ^{2}\omega _{c2}^{2}),\\ c=\omega (2\omega _{12}^{2}\omega _{c1}\Omega _{2}+\omega _{c1}(\omega ^{2}\omega _{c2}^{2}-\Omega _{2}^{2})-\omega _{12}^{4}(\omega _{c1}+\omega _{c2})) ,\\ d=\omega _{12}^{2}(-\Omega _{1}\Omega _{2}+\omega ^{2}\omega _{c1}\omega _{c2} +\omega _{12}^{2}(\Omega _{1}+\Omega _{2})),\\ e=\omega \omega _{12}^{2}(-\omega _{c2}\Omega _{1}-\omega _{c1}\Omega _{2} +\omega _{12}^{2}(\omega _{c1}+\omega _{c2})). \end{array}\right. }$$

By substituting the suitable values as $f=1 \; \text{m/s}^{2}$, $\omega _{c1}=\omega _{c2}=0.01\omega _{2}$, $\omega _{1}=0.9\omega _{2}$ and $\gamma _{1}=\gamma _{2}=0.05\omega _{2}$ into Eq. (19) we obtain the oscillation amplitudes versus the frequency of particles along the x- and z-directions, see Fig. 7c–f. According to Fig. 7c,$\omega _{12}=0.3\omega _{2}$, when we are considering the oscillations of the particles in x direction, coupling between two particles does not take placed well. This can be understood from the different oscillation frequencies of the particles. While we study the oscillations along z direction, see Fig. 7d, the role of the external magnetic field in the coupling between two particles is further revealed so that both particles have the same resonance frequencies. Furthermore, when $\omega _{12}=0$, see Fig. 7e and f, the oscillation amplitudes of $q_{2}$ along both of x- and z-directions are vanished and for $q_{1}$ only one of the peaks is appeared. These results show that the interaction between two particles is canceled.  Therefore, these oscillatory behaviors are in a good agreement with the corresponding ones in the TMOKE signal curves, in both cases $\omega _{12}=0.3\omega _{2}$ ($t_{Ag}=3 \; \text{nm}$) and $\omega _{12}=0$ ($t_{Ag}=20 \; \text{nm}$).

## Influence of different layers on the second resonance of TMOKE signal characteristics

### Effects of replacement of the Co layer with NiFe

**Figure 8:**
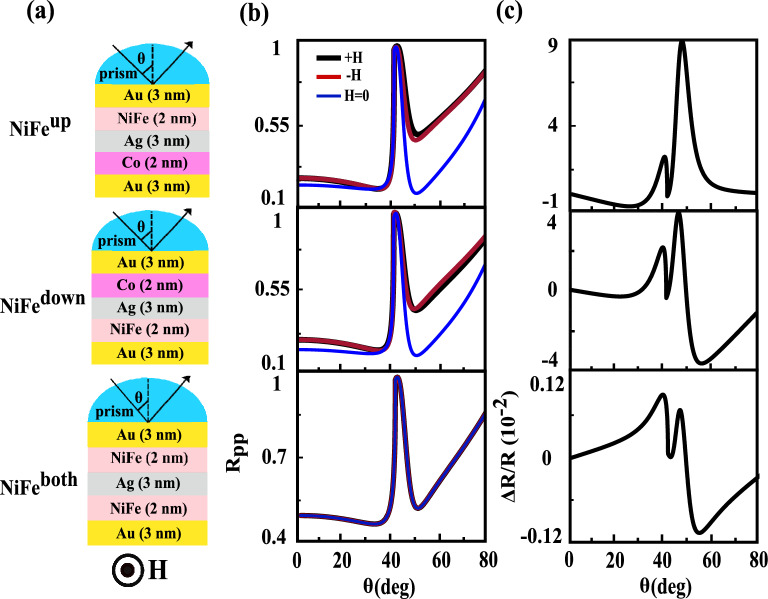
(**a**) schematic of the proposed MO heterostructures in the presence of NiFe layer, $NiFe^{up}$, $NiFe^{down}$ and $NiFe^{both}$ from up to down, respectively. The $R_{PP}(\pm H,H=0)$ curves and their resulting TMOKE signal as a function of incident angle are represented by (**b**) and (**c**), respectively.

**Figure 9:**
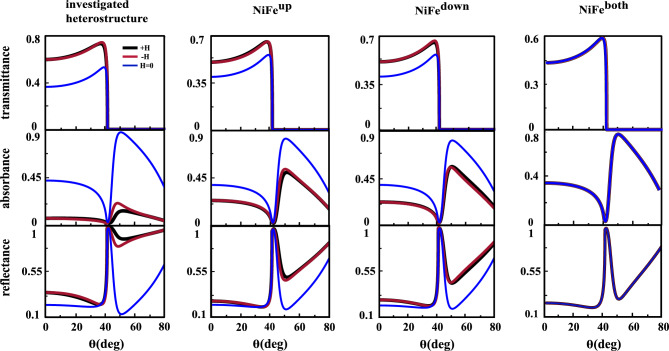
Transmittance, absorbance and reflectance, see from up to down, for investigated heterostructure, $NiFe^{up}$, $NiFe^{down}$ and $NiFe^{both}$, see from left to right, respectively.

In this position, we are interesting to study the influence of replacement of the ferromagnetic layer with another one in the investigated heterostructure. For this purpose, the ultra-soft NiFe with low coercivity and small saturation field was chosen as magnetic layer. The NiFe (81/19) layer has zero magnetostriction and high permeability^51^. At the first step we replace one of the Co layer with NiFe, refractive index $n=2.18+i3.73$ and MO constant $Q=0.0117+i0.0007$^52^), see the top and middle structures of Fig. 8a. In the second step both Co layers are replaced with NiFe. It is shown by the bottom structure of Fig. 8a. This replacement leads to change in the permittivity tensor and thus in the second peak characteristics. The angular dependences of $R_{PP}(\pm H, H=0)$ and their resulting TMOKE signals for three different locations of the NiFe layer which are denoted as $NiFe^{up}$, $NiFe^{down}$ and $NiFe^{both}$ are shown from up to down in Fig. 8b and c, respectively. By comparing angular TMOKE curves in Fig. 8c one can conclude that the magnitude of the second peak for $NiFe^{up}$ is larger than corresponding one for $NiFe^{down}$. Due to the imaginary part of diagonal elements of the NiFe permittivity tensor, that are responsible for absorption in the structure, are smaller than corresponding elements in the Co permittivity tensor more evanescent wave can penetrate from up (Au/NiFe interface) to down (Co/Au interface) of the $NiFe^{up}$ structure. Consequently, the magnitude of second peak increased. Also, by comparing the bottom frames of Figs. 2c and 8c we conclude that the value of second peak for the structure with two Co layers is two order of magnitude larger than that of for $NiFe^{both}$ structure. This obvious difference in the values of second peak is resulting from the real parts of diagonal elements of the Co permittivity tensor are larger than corresponding elements in the permittivity tensor of NiFe. The total reflectance, transmittance and absorbance for investigated heterostructure and structures with NiFe layer are plotted in Fig. 9. The interesting point for all of the structures is that the transmittance becomes zero after angle of the total internal reflection and in this angle the reflectance and absorbance are maximum and minimum, respectively. On the contrary, the maximum of the absorbance occurs exactly at the same angle in which reflectivity becomes minimum (SPP excitation angle).

**Figure 10:**
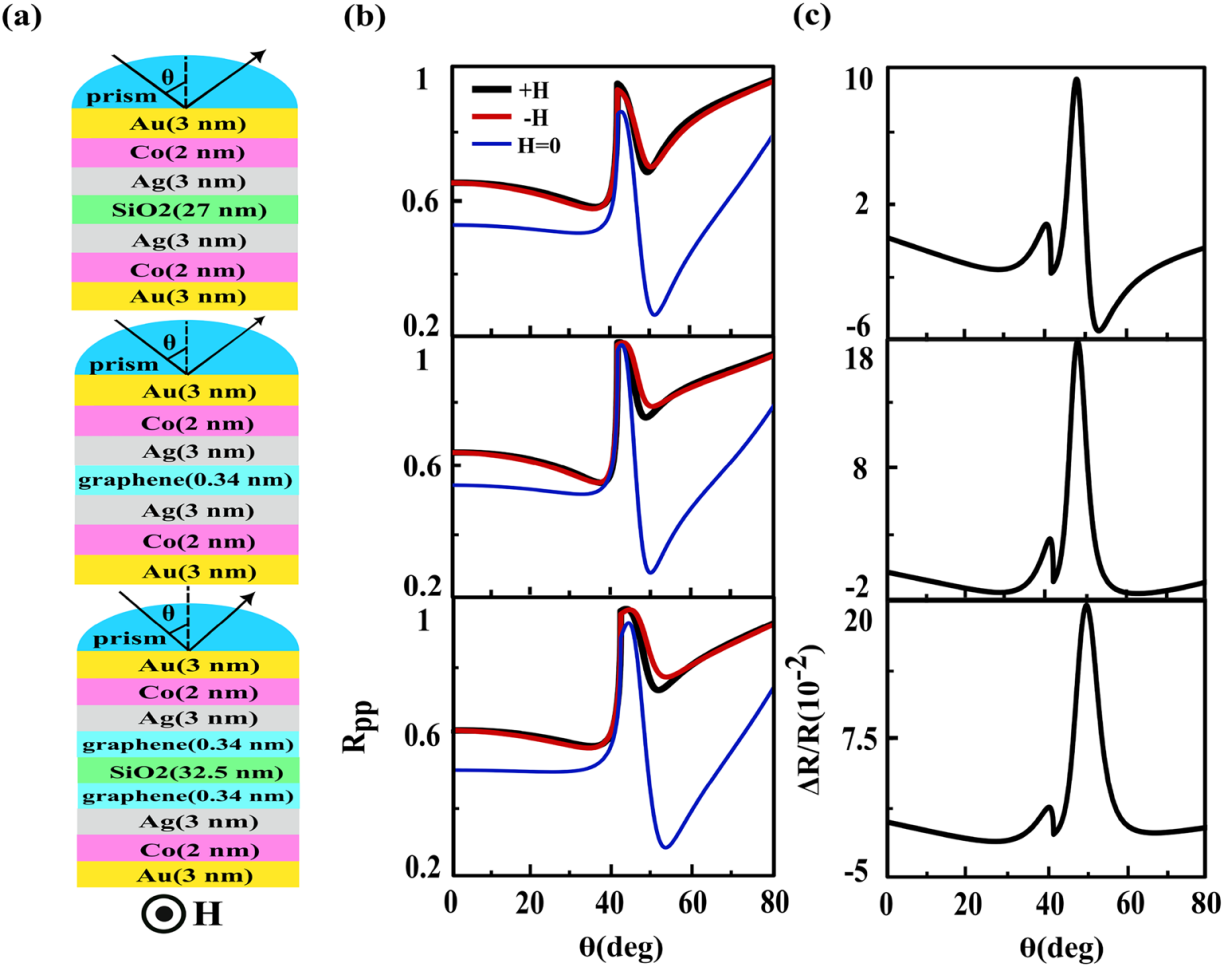
(**a**) schematic of the proposed MO heterostructure in the presence of $\text{SiO}_{2}$, graphene and a combination of both of them from up to down, respectively. (**b**) and (**c**) indicate the reflectivity curves as a function of incident angle in the absence and presence of the external magnetic field and their resulting TMOKE signal, respectively.

### Effects of incorporation of SiO_2_ and graphene spacers

Now, we study the effects of incorporation of different layers on the interaction strength between two MP parts of the investigated heterostructure and finally on the second resonance of TMOKE signal characteristics. Firstly, we intercalate SiO_2_ ($n_{\text{SiO}_{2}}=1.457$^11^) spacer inside the investigated heterostructure such that two trilayer MO structures are above and below of this spacer layer, see the top frame of Fig. 10a. We examine different thicknesses of the spacer and find that the maximum value of second peak occurs at an optimum thickness of 27 nm. Going one step further, we incorporate a graphene monolayer with 0.34 nm thickness instead of the $\text{SiO}_{2}$ layer, see the middle frame of Fig. 10a. The refractive index of graphene in the visible region for this thickness is given by $n=3.0+ic_{1}\lambda /3$ where $\lambda$ is wavelength of the incident light and $c_{1}=5.446 \; \upmu \text{m}^{-1}$^53^. The choice of graphene as separating layer was due to the fact that in the presence of a graphene monolayer, the intensity of SPP considerably enhances^54^.  At the end of this investigation task we insert combination of the $\text{SiO}_{2}$ and graphene spacers into investigated heterostructure, see the bottom frame of Fig. 10a. The reflectivity curves with and without external magnetic field as a function of incident angle and the resulting TMOKE signal for these structures are shown from top to bottom in Fig. 10b and c, respectively. By comparing different parts of Fig. 10c it is clear that in the presence of a graphene monolayer, the magnitude of second peak is larger than corresponding quantity of other proposed heterostructures.

## Theoretical analysis of the sensor response of proposed heterostructures

**Figure 11:**
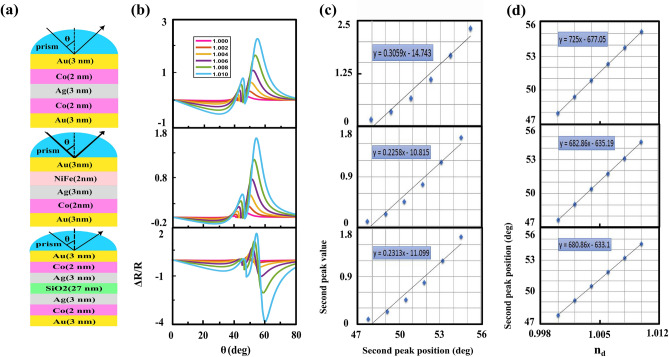
(**a**) Schematic of peroposed heterostructures which we obtained sensitivity for them, (**b**) their corresponding angular TMOKE signal for different values of the refractive index of the sensing medium, (**c**) the value of the second peak as a function of its occurrence angle for each of $n_{d}$ and (**d**) position of the second peak as a function of $n_{d}$. In both (**c**) and (**d**) parts, the blue diamonds correspond to the results of part (**b**), and the black solid line corresponds to a fit to a straight line. The product of slopes obtained from (**c**) an (**d**) parts defines the sensitivity of heterostructures, see Eq. (20).

As we know the plasmonic sensors based on the PIT and fano resonance effects have higher sensitivity than the conventional surface plasmon resonance sensors. Therefore, it is predicted that the appeared second peak in TMOKE signal of MP heterostructures also can improve the performance of the conventional MO refractive index sensors. For calculating sensitivity of the intensity-interrogated sensor which is changed by refractive index of the sensing medium in the Kretschmann configuration, it is better to use the following formula^39,55–57^:

20 $$\begin{aligned} \eta =\frac{\partial S}{\partial n_{d}}=\frac{\partial S}{\partial \theta } \times \frac{\partial \theta }{\partial n_{d}} \end{aligned}$$

where *S* is the sensor signal (either optic or magneto-optic). In the our particular case, *S* is the value of the second peak that occurred at its corresponding angle, $\theta$, for each $n_{d}$. $n_{d}$ is the refractive index of the sensing medium. According to Eq. (20), the sensitivity has two contributions, the slope of the angular TMOKE curve, $\partial S / \partial \theta$ and angular displacements of this curve when the refractive index changes, $\partial \theta / \partial n_{d}$. We change refractive index of the medium surrounding the sensor from $n_{d}=1$ to $n_{d}=1.010$ by step of $\Delta n=0.002$ and extract these two contributions for three types of the our proposed heterostructures, see Fig. 11. The sensitivities of these structures, which are obtained by substituting these two contributions in Eq. (20), are listed in Table 1. We compare these results by data reported in the literatures for conventional MO structures, see Table. 1.

**Table 1:** Comparison between refractive index sensitivity, defined by Eq. (20), of proposed heterostructures and different MO structures reported in literatures.

Structures	$\lambda (\text{nm})$	$\eta (1/RIU)$
Au(3 nm)/Co(2 nm)/Ag(3 nm)/Co(2 nm)/Au(3 nm)	632.8	221.78
Au(3 nm)/NiFe(2 nm)/Ag(3 nm)/Co(2 nm)/Au(3 nm)	632.8	154.19
Au(3 nm)/Co(2 nm)/Ag(3 nm)/$\text{SiO}_{2}(27 \; \text{nm})$/Ag(3 nm)/Co(2 nm)/Au(3 nm)	632.8	157.28
Cr(2 nm)/Au(39.6 nm)/Co(2 nm)/Au(17.8 nm)^57^	633	0.7
Cr(2 nm)/Au(25.3 nm)/Co(4.3 nm)/Au(28 nm)^57^	633	0.62
Cr(2 nm)/Au(38.1 nm)/Co(2 nm)/Au(17.1 nm)^57^	850	4.47
Cr(2 nm)/Au(27.2 nm)/Co(3.9 nm)/Au(28.8 nm)^57^	850	3.81
Au(15 nm)/Co(2 nm)^39^	660	0.2
Au/Co/ single type of Au nanorod^58^	653	33.313
Au/Co/ two types of Au nanorods^58^	653	2.491
Au/Co/ three types of Au nanorods^58^	653	11.847

According to right column of Table  1 it is easy to see that the our proposed heterostructures are more sensitive than conventional MO structures.

## Discussion

We first proposed a symmetric MO heterostructure with two identical MP parts that separated from each other by a noble metallic layer in the Kretschmann configuration. When the separating layer was thick, the angular TMOKE curve of this heterostructure became similar to the corresponding one for the conventional trilayer MO structure. By decreasing the thickness of this layer, the interaction between two MP portions increased as a consequent, a new peak appeared in the TMOKE signal compared to the previous case. We introduced this new peak as a second resonance of TMOKE signal. We also studied the effects of the replacement of magnetic layer with another one and insertion of different spacers between two MP branches of the investigated heterostructure. In order to elucidate the occurrence mechanism of the second resonance we obtained dispersion relation of the basic heterostructure and plotted it. For small thicknesses of the spacer, the dispersion diagram exhibitted four branches which correspond to the SPP excitation at the four interfaces of the heterostructure. This behavior shows the coupling between magneto-plasmon modes of the two MP components. Gradually, as the thickness of spacer increases, the interaction between two MP portions decreases and both magneto-plasmon modes uncoupled so that only two upper branches are remained. Furthermore, we calculated the electric field distribution along the SPP propagation at the angle of second peak and compared it with the corresponding distribution at the angle of maximum TMOKE signal for the conventional structure and proposed heterostructure with thick separating layer. This task demonstrated that the nature of second peak was completely different from two another maximums. We also established a perfect analogy between investigated heterostructure and the coupled oscillators model. This analogy allows us to conclude that the appearance of the second peak in the TMOKE signal is direct result of the coupling between MO activities of the both MP parts. By simulation we demonstrated that the coupling between two magneto-plasmon modes of the upper and lower MP parts causes the sensitivity of the refractive index sensors significantly increases compared to the corresponding conventional MO sensors based on the TMOKE signal.

## Methods

### Fabrication process

**Figure 12:**
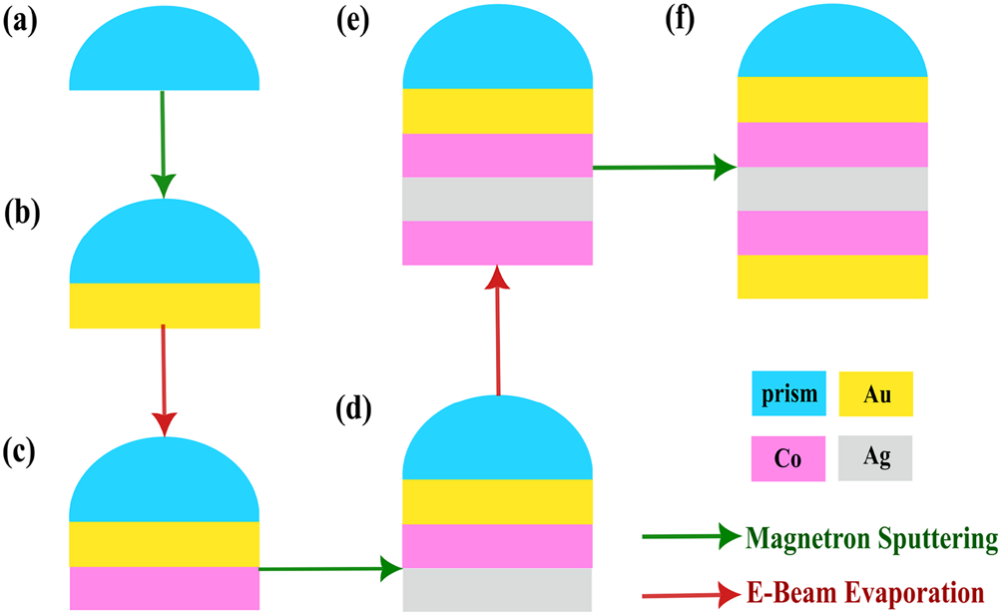
Suggested fabrication process flow for the first proposed MP heterostructure.

There are no fabrication challenges for construction of the proposed heterostructuers. For example, the fabrication steps of the first proposed heterostructure are depicted in different parts of Fig. 12.

### Supplementary Information

Supplementary Information.

## Data Availability

The calculated results during the current study are available from the corresponding author on reasonable request.
