# Supplementary material for: Magneto-optical heterostructures with second resonance of transverse magneto-optical Kerr effect
Source: Sci Rep. 2024 Feb 12;14:3493. doi: 10.1038/s41598-024-54039-3 (PMC10861523; doi:10.1038/s41598-024-54039-3)
Supplement: Supplementary file 1 — Supplementary Information. [file 41598_2024_54039_MOESM1_ESM.docx]

Magneto-optical heterostructures with second resonance of transverse
magneto-optical Kerr effect

Amene Rezaeian and Mahmood Hosseini Farzad

**Explanation of second resonance of TMOKE by dispersion relation of the investigated heterostructure**

Now we are interesting to apply some mathematical manipulation in order to obtain the characteristic equation of this MO heterostructure. For this task we write the electric and magnetic field components of a TM mode which are obtained by using Maxwells and wave equations in different regions of the heterostructure, see Figure. S1 (a). For the first Au layer, $z>0$, the fields are:

$$H_{y}=A_{1}e^{i\beta x}e^{-k_{1}z}$$

$E_{x}=\frac{ik_{1}A_{1}}{w\varepsilon_{0}\varepsilon_{1}}e^{i\beta x}e^{-k_{1}z}$

$E_{z}=-\frac{\beta A_{1}}{w\varepsilon_{0}\varepsilon_{1}}e^{i\beta x}e^{-k_{1}z},$ (1)

For the first Co layer, 0 $> z > -b$, we get:

$H_{y}=A_{2}e^{i\beta x}e^{+k_{d}z}+A_{3}e^{i\beta x}e^{-k_{d}z}$

$E_{x}=-\frac{iA_{2}\left( k_{m}\varepsilon_{m}+g\beta\right)}{w\varepsilon_{0}\left( \varepsilon_{m}^{2}-g^{2} \right)}e^{i\beta x}e^{+k_{m}z}+\frac{iA_{3}\left( k_{m}\varepsilon_{m}+g\beta\right)}{w\varepsilon_{0}\left( \varepsilon_{m}^{2}-g^{2} \right)}e^{i\beta x}e^{-k_{m}z}$

$E_{z}=+\frac{A_{2}\left( \beta\varepsilon_{m}+k_{m}g \right)}{w\varepsilon_{0}\left( \varepsilon_{m}^{2}-g^{2} \right)}e^{i\beta x}e^{+k_{m}z}+\frac{A_{3}\left( \beta\varepsilon_{m}+k_{m}g \right)}{w\varepsilon_{0}\left( \varepsilon_{m}^{2}-g^{2} \right)}e^{i\beta x}e^{-k_{m}z},$ (2)

For the middle (Ag) layer, *-β    -β - *, we have:

$H_{y}=A_{4}e^{i\beta x}e^{+k_{2}z}+A_{5}e^{i\beta x}e^{-k_{2}z}$

$E_{x}=-\frac{ik_{2}A_{4}}{w\varepsilon_{0}\varepsilon_{2}}e^{i\beta x}e^{+k_{2}z}+\frac{ik_{2}A_{5}}{w\varepsilon_{0}\varepsilon_{2}}e^{i\beta x}e^{-k_{2}z}$

$E_{z}=+\frac{\beta A_{4}}{w\varepsilon_{0}\varepsilon_{2}}e^{i\beta x}e^{+k_{2}z}+\frac{\beta A_{5}}{w\varepsilon_{0}\varepsilon_{2}}e^{i\beta x}e^{-k_{2}z},$ (3)

For the second Co layer, *-β -     -β -* 2*τ*, we give:

$H_{y}=A_{6}e^{i\beta x}e^{+k_{d}z}+A_{7}e^{i\beta x}e^{-k_{d}z}$

$E_{x}=-\frac{iA_{6}\left( k_{m}\varepsilon_{m}+g\beta\right)}{w\varepsilon_{0}\left( \varepsilon_{m}^{2}-g^{2} \right)}e^{i\beta x}e^{+k_{m}z}+\frac{iA_{7}\left( k_{m}\varepsilon_{m}+g\beta\right)}{w\varepsilon_{0}\left( \varepsilon_{m}^{2}-g^{2} \right)}e^{i\beta x}e^{-k_{m}z}$

$E_{z}=+\frac{A_{6}\left( \beta\varepsilon_{m}+gk_{m} \right)}{w\varepsilon_{0}\left( \varepsilon_{m}^{2}-g^{2} \right)}e^{i\beta x}e^{+k_{m}z}+\frac{A_{7}\left( \beta\varepsilon_{m}+gk_{m} \right)}{w\varepsilon_{0}\left( \varepsilon_{m}^{2}-g^{2} \right)}e^{i\beta x}e^{-k_{m}z},$ (4)

Finally for the last (Au) layer, *-*2*β -   *, we get:

$$H_{y}= A_{8}e^{i\beta x}e^{+k_{1}z}$$

$E_{x}=\frac{ik_{1}A_{8}}{w\varepsilon_{0}\varepsilon_{1}}e^{i\beta x}e^{+k_{1}z}$

$E_{z}=\frac{-\beta A_{8}}{w\varepsilon_{0}\varepsilon_{1}}e^{i\beta x}e^{+k_{1}z}.$ (5)

The continuity of $H_{y}$ and $E_{x}$ at the boundaries of * *= 0, * *= *-β*, * *= *-β -  *and * *= *-*2*β -  *leads to the following matrix notation

$\left( \begin{matrix} A & B \\ C & D \end{matrix} \right)\times\left( \begin{matrix} E \\ F \end{matrix} \right)=\left( \begin{matrix} 0 \\ 0 \end{matrix} \right),$ (6)

where the parameters $A$, $B$, $C$, $D$, $E$ and $F$ are defined as:

$$A=\left( \begin{matrix} 1 & -1 & -1 & 0 \\ \frac{k_{1}}{\varepsilon_{1}} & \frac{k_{m}\varepsilon_{m}+\beta g}{\varepsilon_{m}^{2}-g^{2}} & -\frac{k_{m}\varepsilon_{m}+\beta g}{\varepsilon_{m}^{2}-g^{2}} & 0 \\ 0 & e^{-k_{m}b} & e^{k_{m}b} & -e^{-k_{2}b} \\ 0 & -\frac{k_{m}\varepsilon_{m}+\beta g}{\varepsilon_{m}^{2}-g^{2}}e^{-k_{m}b} & \frac{k_{m}\varepsilon_{m}+\beta g}{\varepsilon_{m}^{2}-g^{2}}e^{k_{m}b} & \frac{k_{2}}{\varepsilon_{2}}e^{-k_{2}b} \end{matrix} \right),$$

$$B=\left( \begin{matrix} 0 & 0 & 0 & 0 \\ 0 & 0 & 0 & 0 \\ -e^{+k_{2}b} & 0 & 0 & 0 \\ -\frac{k_{2}}{\varepsilon_{2}}e^{+k_{2}b} & 0 & 0 & 0 \end{matrix} \right),$$

$$C=\left( \begin{matrix} 0 & 0 & 0 & e^{-k_{2}(b+t)} \\ 0 & 0 & 0 & -\frac{k_{2}}{\varepsilon_{2}}e^{-k_{2}(b+t)} \\ 0 & 0 & 0 & 0 \\ 0 & 0 & 0 & 0 \end{matrix} \right),$$

$$D=\left( \begin{matrix} e^{+k_{2}(b+t)} & -e^{-k_{m}(b+t)} & -e^{k_{m}(b+t)} & 0 \\ \frac{k_{2}}{\varepsilon_{2}}e^{+k_{2}(b+t)} & \frac{k_{m}\varepsilon_{m}+\beta g}{\varepsilon_{m}^{2}-g^{2}}e^{-k_{m}(b+t)} & -\frac{k_{m}\varepsilon_{m}+\beta g}{\varepsilon_{m}^{2}-g^{2}}e^{k_{m}(b+t)} & 0 \\ 0 & e^{-k_{m}(2b+t)} & e^{k_{m}(2b+t)} & -e^{-k_{2}(2b+t)} \\ 0 & -\frac{k_{m}\varepsilon_{m}+\beta g}{\varepsilon_{m}^{2}-g^{2}}e^{-k_{m}(2b+t)} & \frac{k_{m}\varepsilon_{m}+\beta g}{\varepsilon_{m}^{2}-g^{2}}e^{k_{m}(2b+t)} & -\frac{k_{1}}{\varepsilon_{1}}e^{-k_{1}(2b+t)} \end{matrix} \right),$$

$E=\left( \begin{matrix} A_{1} \\ A_{2} \\ A_{3} \\ A_{4} \end{matrix} \right)$and

$F=\left( \begin{matrix} A_{5} \\ A_{6} \\ A_{7} \\ A_{8} \end{matrix} \right)$.

To have a nontrivial solution, the determinant of the coefficients matrix must be zero that yields the following SPP dispersion relation:

$tanh( k_{2}t)=\frac{-2\alpha\sigma}{\sigma^{2}+\alpha^{2}} ,$ (7)

where the parameters *α* and *σ* are defined as:

$\alpha=\frac{k_{2}}{\varepsilon_{2}}(1+\frac{k_{1}}{\varepsilon_{1}} \frac{\varepsilon_{m}^{2}-g^{2}}{k_{m}\varepsilon_{m}+g\beta}\tanh k_{m}b),$

$$\sigma=\frac{k_{1}}{\varepsilon_{1}}(1+\frac{\varepsilon_{1}}{k_{1}} \frac{k_{m}\varepsilon_{m}+g\beta}{\varepsilon_{m}^{2}-g^{2}}\tanh k_{m}b).$$

Figure. S1 (b) shows the dispersion diagram of the investigated heterostructure which is obtained by substituting the quantities of $\varepsilon_{i}=1-\frac{\omega_{pi}^{2}}{\omega^{2}}, k_{i}=\sqrt{\beta^{2}-k_{0}^{2}\varepsilon_{i}} , i=1,2$,

$\varepsilon_{m}=1-\frac{\omega_{pm}^{2}}{\omega^{2}}$ , $k_{m}=\sqrt{\beta^{2}-\frac{k_{0}^{2}}{\varepsilon_{m}}\left( \varepsilon_{m}^{2}-g^{2} \right)}$ and $g=\frac{\omega_{pm}^{2}\omega_{c}}{\omega(\omega^{2}+\omega_{c}^{2})}$ into Eg. (7). Where $\omega_{p1}$, $\omega_{p2}$ and $\omega_{pm}$ are the plasma frequencies of the Au, Ag and Co layers, respectively. $\omega_{c}={eB}/m$ is the cyclotron frequency. $e$ and $m$ are the charge and mass of electron. $B$ is the external magnetic field. The basic four dispersion curves correspond to the SPP excitation at the Au/Co, Co/Ag, Ag/Co and Co/Au interfaces of the heterostructure, from up to down, respectively. As it can be observed in Figure. S1 (b), by increasing the thickness of Ag layer the coupling between upper and lower SPPs decreases. For a thickness of 120 nm, this coupling is completely canceled and two upper branches of dispersion curve remain. These behaviors confirm the results obtained from the simulations discussed above. In the limit as *β* goes to infinity, Eq. (7) is simplified to the following equation:

$\left( \varepsilon_{1}+\varepsilon_{m}-g \right)\left( \varepsilon_{2}+\varepsilon_{m}-g \right)=0$ (8)

After substituting the parameters $\varepsilon_{1}$, $\varepsilon_{2}$, $\varepsilon_{m}$ and $g$ into Eq. (8), it splits into two equations as follow:

$2\omega^{4}+\omega^{2}\left( 2\omega_{c}^{2}{-\omega}_{p1}^{2}-\omega_{pm}^{2} \right)-\omega\omega_{c}\omega_{pm}^{2}-\omega_{c}^{2}(\omega_{p1}^{2}+\omega_{pm}^{2})$=0,

$2\omega^{4}+\omega^{2}\left( 2\omega_{c}^{2}{-\omega}_{p2}^{2}-\omega_{pm}^{2} \right)-\omega\omega_{c}\omega_{pm}^{2}-\omega_{c}^{2}(\omega_{p2}^{2}+\omega_{pm}^{2})$= 0. (9)

As it is shown from Figure. S1 (b), each two branches of the dispersion curve merge to each other and consequently only two horizontally lines are observed for very large value of *β*. These lines are obtained from the solutions of Eq. 9.


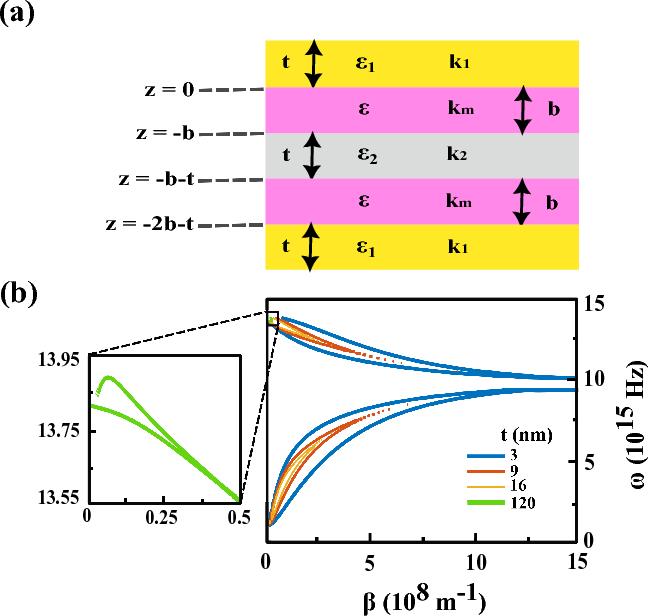


Figure S1. (a) schematic of our interested heterostructure with different regions. $\varepsilon_{1}$, *ε* and $\varepsilon_{2}$ are dielectric constants of Au, Co and Ag layers, respectively. $k_{1}$, $k_{m}$ and $k_{2}$ are their corresponding z-component wave vectors. t and b are thicknesses of the noble metallic and Co layers which are equal to 3 nm and 2 nm, respectively in our calculations. (b) dispersion diagrams for the proposed heterostructure with 3 nm, 9 nm, 16 nm and 120 nm of Ag layer thicknesses. The inset magnifies the dispersion curve of the heterostructure when the thickness of Ag layer is equal to 120 nm.
